# A Cardinality Estimator in Complex Database Systems Based on TreeLSTM

**DOI:** 10.3390/s23177364

**Published:** 2023-08-23

**Authors:** Kaiyang Qi, Jiong Yu, Zhenzhen He

**Affiliations:** 1 School of Software, Xinjiang University, Urumqi 830091, China; 2 College of Information Science and Engineering, Xinjiang University, Urumqi 830046, China

**Keywords:** cardinality estimation, tree long short-term memory, query optimization, deep learning

## Abstract

Cardinality estimation is critical for database management systems (DBMSs) to execute query optimization tasks, which can guide the query optimizer in choosing the best execution plan. However, traditional cardinality estimation methods cannot provide accurate estimates because they cannot accurately capture the correlation between multiple tables. Several recent studies have revealed that learning-based cardinality estimation methods can address the shortcomings of traditional methods and provide more accurate estimates. However, the learning-based cardinality estimation methods still have large errors when an SQL query involves multiple tables or is very complex. To address this problem, we propose a sampling-based tree long short-term memory (TreeLSTM) neural network to model queries. The proposed model addresses the weakness of traditional methods when no sampled tuples match the predicates and considers the join relationship between multiple tables and the conjunction and disjunction operations between predicates. We construct subexpressions as trees using operator types between predicates and improve the performance and accuracy of cardinality estimation by capturing the join-crossing correlations between tables and the order dependencies between predicates. In addition, we construct a new loss function to overcome the drawback that *Q-error* cannot distinguish between large and small cardinalities. Extensive experimental results from real-world datasets show that our proposed model improves the estimation quality and outperforms traditional cardinality estimation methods and the other compared deep learning methods in three evaluation metrics: *Q-error*, *MAE*, and *SMAPE*.

## 1. Introduction

The query optimizer is a crucial part of DBMSs designed to analyze and optimize SQL queries to improve query performance. One of the critical issues in query optimization is the cardinality estimation, the main task of which is to estimate the number of tuples generated by a (sub)query [1,2]. An accurate estimation result can guide the query optimizer to choose the right query plan and help improve query performance. Despite the importance of the cardinality estimation problem, researchers generally agree that it has still not been fully solved [3,4,5]. Open-source and commercial database management systems typically produce $10^{4}$ to $10^{8}$ estimation errors when a query involves a large amount of data [3,6]. Previous research has shown that traditional cardinality estimation methods are typically based on models with strong assumptions (e.g., data independence and consistency). These assumptions can be affected by the data distribution, leading to slower queries and larger estimation errors. The fundamental reason for the large estimation error is the inability of the model to capture the relationship between table-to-table join operations. Accurately capturing the relationships between different tables or columns directly determines the quality of our proposed method. To address this problem, we designed a new deep learning method called TreeLSTM. Deep learning excels in cardinality estimation because the neural network can learn and be trained in how to obtain hidden relationships between samples and can predict new data based on the learned knowledge while continuously improving the estimation accuracy as the model is trained and optimized.

With the development of machine learning techniques, researchers have also been using machine learning in recent years to address the problem of inaccurate cardinality estimation [6,7,8,9,10]. The multi-set convolutional neural network (MSCN) [7] is the current state-of-the-art method. The MSCN uses a new deep learning method to estimate cardinality that fully considers the characteristics of samples, multi-table joins, and predicates. However, the MSCN can only solve simple equivalent join operations, does not support operators, and cannot represent complex structures, such as tree query plans using average-pooling neural networks.

There are usually several challenges in designing an effective cardinality estimator. Firstly, a model needs to be designed for cardinality estimation. Secondly, the model should be able to handle complex SQL queries, where, typically, a complex query may contain multiple table join operations and an arbitrary number of predicates connected by Boolean operators. Thirdly, the model should have a strong generalization capability to support various queries. The last challenge concerns the loss function. The Q_error_loss is commonly used in cardinality estimation [7]. The *Q-error* is a symmetric metric used to calculate the ratio of the estimated value to the true value, and it provides a relative error. However, the *Q-error* has an apparent disadvantage with large and small cardinality problems. In simple terms, if the true and estimated cardinalities are equal to 100 and 10, respectively, the *Q-error* will be 10. The same *Q-error* will be obtained when the true and the estimated cardinalities are equal to 10,000 and 5000, respectively. At this point, we cannot accurately estimate the cardinality value. Our contributions of in this study are summarized as follows:

1. We propose a cardinality estimation model based on deep learning for the tree long short-term memory neural network. Moreover, we added a sampling method to the model to solve the problem that the sampling-based method increases the spatial overhead and the 0-tuple problem [11]. Meanwhile, we adopted the idea of natural language processing to encode the SQL queries so that the model can capture the semantics implied in the queries. We also constructed the “WHERE” clause as a tree structure and generate tree vectors based on the operators between predicates. Thus, the model can capture the multiple tables’ join relationships and the sequential dependencies between predicates in a complex query;
2. In order to solve the problem mentioned above that the *Q-error* cannot distinguish between large and small cardinalities, we designed a hybrid loss function that combines two different losses; namely, *Q_error_loss* and absolute value loss. This loss function is based on the idea of the *Q-error* and absolute error and uses an adjustable parameter α to balance the contributions of the two loss functions. The *Q-error* part optimizes the relative error, while the absolute error part distinguishes between the large and small cardinalities problems;
3. In addition, we evaluated our model with two large datasets, the IMDB [3] and DMV [12] datasets, to demonstrate the generalization of our model. The experimental results show that our method outperforms traditional and other learning-based cardinality estimation methods. They also demonstrate the feasibility and superiority of our proposed model.

The remainder of this paper is organized as follows. Section 2 summarizes the related cardinality estimation work, including traditional and learning-based cardinality estimation methods. Section 3 describes the working mechanism of our proposed deep learning model. In Section 4, we describe extensive experiments on the cardinality estimation model of TreeLSTM and evaluate and analyze the experimental results. Section 5 concludes this paper and provides an outlook on future research directions.

## 2. Related Work

Cardinality estimation has been studied for decades as an important aspect of traditional database query optimization. In the following, we introduce the existing techniques for traditional and learning-based cardinality estimation, as shown in Table 1.

### 2.1. Traditional Cardinality Estimation Methods

The current traditional cardinality estimators in production environments still suffer from significant errors, which can reach several orders of magnitude. In this paper, we classify traditional cardinality estimation methods into two categories.

1. Summary-based methods. These methods collect statistical information about the database in advance and make independent assumptions to estimate cardinality quickly. For example, the histogram-based method [13,14,15] targets real databases where the distribution of each column value is not uniform, so each column in the data table is summarized as an equal-width or equal-depth histogram to keep information about each data range to fit the real distribution of the data. The implementation of this method is relatively simple and it is widely used, but it is ineffective when estimating the correlation between different columns. Data sketching [16,17,18,19] is also a summary-based cardinality estimation method, the core idea of which is to use a hash function to map a tuple value to a set of positions on a bitmap and to add count values to the corresponding positions according to the number of tuple values. The cardinality number of the tuple values can be inferred by counting the number of consecutive zeros or hits in the bitmap. This method focuses more on saving memory and improving estimation robustness, but the method does not apply to estimating range queries.
2. Sampling-based methods [20,21]. The core idea of these methods is to randomly select a certain proportion or a certain number of tuples from the original data table and then to estimate the cardinality of the query in the original database based on the size of the result after executing the query on the sampled set divided by the corresponding scaling ratio. The effectiveness of this method is influenced by the sampling strategy, and it is more effective if the sampling strategy can better reflect most of the data distribution. However, due to the random nature of sampling, the method may produce results that do not match the sampled set, resulting in large estimation errors.

### 2.2. Learning-Based Cardinality Estimation Methods

Learning-based methods differ from traditional cardinality estimation methods in that they can learn correlations between multiple columns and tables. Learning-based methods can be classified as data-driven and query-driven.

1. Data-driven cardinality estimation: This is a class of unsupervised methods that aim to approximate the cardinality of the joint data distribution [22,23]. In [24], the authors used the kernel density estimation method to estimate cardinality. The core idea is to sample some tuples in data tables randomly, and the closer the query and the sampled tuples are, the more likely it is that the query has a result. The authors used a Gaussian kernel and learnable parameters to control the dispersion of the probability density. In a later study [25], the authors extended the kernel density estimation approach to cardinality estimation of multiple table joins. In [26], the authors used the DeepDB model to capture joint probability distributions for data and essential features, such as correlations across attributes and data distributions for individual attributes. An important difference between DeepDB and other approaches is that it supports direct updates; i.e., insertions, updates, and deletions in the underlying database can be directly absorbed by the model without retraining it. The neural optimizer (Neo) was proposed in [27], which relies on deep neural networks to generate query execution plans. Neo starts its query optimization model from an existing optimizer and continues to learn from incoming queries, building on its successes and learning from its failures. The estimators Naru [28], Neurocard [29], and MADE [30] approximate the data distribution in a table or joint table using a deep autoregressive model, which captures the data distribution in a table by multiplying the estimated data distributions in each column, with an implicit assumption that each column depends on the previous column.
2. Query-driven cardinality estimation: This method was also used in our experiments. Unlike the data-driven approach, the query-driven approach can be considered a regression problem that maps “query” to “cardinality values”, where the labels are the true cardinality values. The main difficulty of this approach lies in the acquisition of training data and the representation of query statements. In [31], a “Black-Box” method was proposed to estimate the query cardinality values. This method provides accurate estimates by grouping queries into grammar families and learning the cardinality distribution of the group directly from the points in the high-dimensional input space without knowing the query execution plan and data distribution. Kipf et al. [7] proposed a method called the multi-set convolutional neural network (MSCN), which decomposes the query into three parts (table names, joins, and predicates) and uses a neural network to process these three inputs independently. The MSCN can model joins and predicates from multiple tables and cover data correlations. However, the approach cannot handle complex query statements and string types in predicates. In [9], the authors proposed an end-to-end learned estimation model for processing complex SQL queries and estimating the cardinality and cost of the queries. The model relies on the DBMS optimizer to generate a physical execution plan for the query. However, obtaining the physical execution plan is difficult, and the model is more complex.
3. Hybrid cardinality estimation: In addition, some current research has attempted to capitalize on the strengths of both types of methods by proposing hybrid approaches. The authors of [32] proposed a deep autoregressive model (UAE) to bridge the gap between query-driven and data-driven approaches. The approach uses data as unsupervised information and the query workload as supervised information for cardinality estimation. Dutt and his team [33] merged a learning-based approach with the cardinality estimation of histograms by using the output of the histogram estimation as an additional feature input. They proposed a design for cardinality estimation using machine learning and an integrated tree-based learning model. However, neither of the hybrid cardinality estimations can be trained directly from the data and the cost of the model is increased.

In recent years, deep learning has made meaningful progress in various fields, including natural language processing, computer vision, and speech recognition. Combining deep learning and database technology is a new research direction aiming to improve database performance and efficiency. This combination demonstrates the promising prospect of using artificial intelligence database technology to improve data performance. Applying deep learning to database technology can harness the power of deep learning to optimize database performance and efficiency to meet the needs of modern data applications better.

## 3. Cardinality Estimator

**Figure 1:**
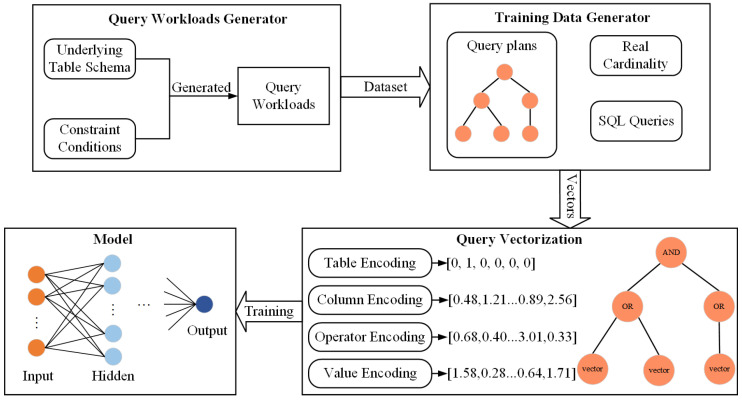
Overview of cardinality estimator.

Our study aims to encode the query statements and then input the encoding into our model to generate an estimated cardinality value. Moreover, we compare the estimated value with the true value to train the model so that our model can be used as a cardinality estimator for new queries. The specific flow of our work is shown in Figure 1 and mainly includes query workload generation, training data generation, feature extraction and encoding, and model training. In general, our task is to build a supervised learning model. Firstly, we need to solve the “cold start problem”; i.e., how to obtain the initial training set. Secondly, we need to consider how to effectively represent the semantic information of the query statements; i.e., how to encode the features of the query statements. Finally, we need to design an efficient model for cardinality estimation. Next, we address these issues and discuss the key technique of our approach, which involves constructing the “WHERE” clause of a query statement as a tree structure so that our model can handle complex queries.

### 3.1. Cold Start Problem

In artificial intelligence for database (AI4DB) research, all learning-based algorithms face a crucial problem, the “cold start problem”, which concerns how to train the model before obtaining the SQL queries. We do this by generating random queries based on the schema information and constraints of the tables and execute the queries in the database to obtain the true cardinality.

The SQL queries for multi-table and single-table datasets are generated similarly, so we use the IMDB dataset as an example. We select six candidate tables in the IMDB dataset and determine their primary and foreign key relationships to generate join conditions. To generate queries, we randomly select join conditions and limit the number of join operations in each query to a maximum of five to reduce the combination space and avoid the combination explosion problem. This approach can better simulate the real data situation and improve the accuracy and generalization of cardinality estimation. For the predicate part, we randomly choose columns of the numeric or string type from the relevant tables and use {>, >=, <, <=, =, !=} to construct selection conditions for logical relations to generate atomic predicates. Then, we aggregate the generated atomic predicates using the “AND” or “OR” operators to generate the complete query statement. Finally, we execute the generated query statements in the database to obtain the true cardinality and delete the zero-cardinality query statements. In this way, we obtain the initial training data.

### 3.2. Representation of SQL Query

**Figure 2:**
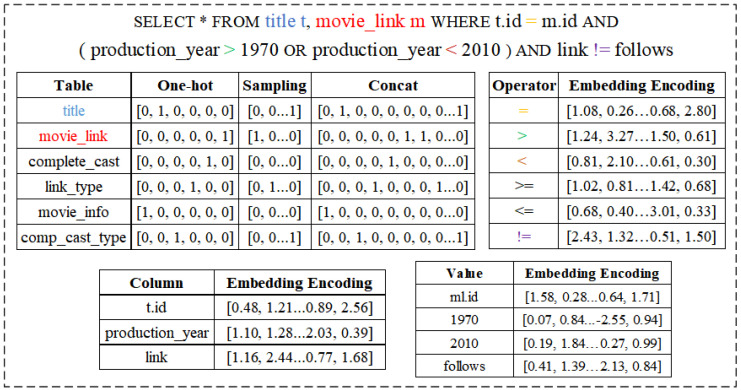
Overview of cardinality estimator (The asterisk in the figure indicates retrieval of all columns from the tables).

This section presents the proposed encoding framework, as shown in Figure 2. We first use a one-hot vector to represent the table name in the query. We then sample the tables and use a bitmap to represent them. Next, we merge the table vector and the bitmap to get the information vector of the tables in the query. Finally, for the join predicates and base table predicates in a query, we use word embedding [34] to transform them into word vectors and construct the “WHERE” clause as a tree structure.

#### 3.2.1. Table Information Encoding

We represent all queries as *Q* and all tables as *T*. Given a query q∈Q, the tables it relates to can be represented by the set $T_{q}$. In a query, the semantics of table names can be seen as relatively independent concerning join predicates and base table predicates. Therefore, the table names can be encoded separately. We first compute the number of tables in the set $T_{q}$ as |$T_{q}$|. The table names are then represented as a unique one-hot vector of length |$T_{q}$| that uniquely identifies a particular table.

To give the model a better understanding of the data distribution in the tables, we encode not only the table names but also the sample tuples of the relevant columns and use a sample bitmap to represent the positions of the eligible samples. The sample bitmap is a vector of length 1000, where each bit indicates whether the sample matches the predicate. If the sample matches the predicate, the corresponding position is 1; otherwise, it is 0. Adding sampled features makes it easier for our model to perform join estimation and learn more information from the table. While traditional sampling methods can improve the accuracy of cardinality estimation, they also increase space expenses. In addition, the traditional method has the limitation that when the query is sparse, the sample is invalid if the bitmap equals zero. This problem greatly affects the estimation accuracy. We use deep learning techniques in our proposed TreeLSTM model, which can handle the 0-tuple problem well by integrating sampling methods. This method can better use the sampling information and improve cardinality estimation accuracy.

#### 3.2.2. Predicate Information Encoding

A neglected problem in cardinality estimation is the disappearance of semantic propagation. Calculating the correct base given a filter condition is easy for single-table queries. However, for multi-table queries, if there are multiple join conditions or the query is complex, the search space becomes huge, leading to gradual semantic disappearance. To meet this challenge, we must choose a vector representation that has memory capabilities or the ability to map words to context-sensitive vectors.

In a query statement, the predicates in the “WHERE” clause are usually a collection of filtering or concatenation conditions. The predicate can contain a join condition, like “t.id = m.id”, or a filter condition, like “production_year > 1980”. In addition to atomic predicates for single conditions, there may be compound predicates, like “production_year > 1980 OR production_year < 2012”. Predicates can help us filter the tuples that match the requirements more precisely and thus get more accurate query results.

The atomic predicates are triples of the form (*col*, *op*, *val*), where *col* is the column name; *op* is the operator, including {>, >=, <, <=, =, !=}; and *val* is the operand. In most previous studies [4,35,36], column names and operators are usually identified using one-hot encoding. Moreover, the operands in these works are numeric data, and string-type data are not supported. Therefore, these studies normalize the operands directly using the maximum and minimum values of each column. However, when it comes to string data, using one-hot encoding may lead to the problem of dimensionality explosion. In particular, when a column in a table has many different values, it will affect the computational efficiency and storage space, and the information in one-hot vectors will be sparse and unable to describe the similarity and semantics between words. To solve this problem, we use the word embedding method to learn the vector representation of predicates. We encode column names, operators, and operands separately as word vectors and then concatenate them to obtain an atomic predicate vector. This approach maps words into a low-dimensional vector space, allowing words with similar semantics to be closer together in the vector space. Overall, this approach can better capture the semantic relationships between predicates and better handle numeric and string operands. As shown in Figure 2, an atomic predicate vector is a concatenation of a column vector, an operator vector, and an operand vector. In addition, for the logical operators in the query (in this study, we only consider “&” and “|”), we do not encode them. We use minimum pooling and maximum pooling instead of “&” and “|”, as described in Section 3.3.3.

#### 3.2.3. Construction of SQL Tree

**Figure 3:**
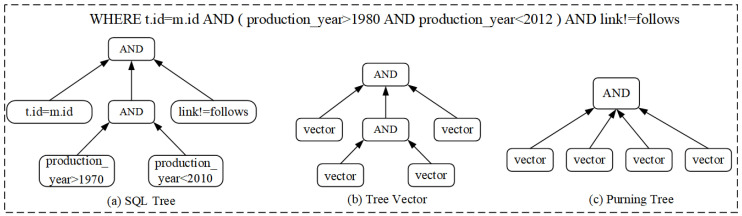
Construction of SQL tree.

In our study, we apply the idea of natural language processing (NLP) to SQL statements, which contain certain semantic information. For example, an SQL statement will have a contextual relationship between the predicate and the conjunction “AND” or “OR”. However, this relationship is not as strong as in NLP. In order to better learn the semantics implied in the query, we construct the query as a tree structure. Specifically, for compound predicates, we first construct an SQL tree, as shown in Figure 3a, based on the prefix or suffix expressions of the “WHERE” clause, generate a vector for each atomic predicate, and then convert the leaf nodes of the SQL tree to the corresponding vectors using a one-to-one mapping strategy. In this way, we generate the tree vectors shown in Figure 3b.

In addition, since the order of joins between predicates in a query statement does not affect the output, we prune the SQL tree to simplify its structure. The pruning operation keeps the predicates in a query statement in the same arrangement as the allowed predicates. As shown in Figure 3c, we construct an equivalent tree structure by pruning the nodes with the same join operator in the SQL tree; i.e., a pruning tree. The characteristic of this pruning tree is that it ensures that all children of a node form a set, and changes in the order of the children in the set do not affect the final output. This feature can simplify the structure of the predicate tree and improve its efficiency and accuracy. Specifically, if multiple predicate nodes have the same join operator, we prune the operators among the children nodes and then traverse from the leaf nodes to the root to merge these nodes. Furthermore, to verify the effectiveness of our pruned tree, we experimentally validate it in Section 4.6.

Algorithm 1 is a description of our proposed pruning tree algorithm. The core idea of the algorithm is to read operands and operators one by one from the input suffix logic expressions. When an operand is encountered, it is encapsulated as a node and pushed onto the stack. When an operator is encountered, two nodes are popped from the stack as the left and right children of that operator, and they are added to the pruning tree as children of the new node. This is repeated until the entire postfix expression is traversed and, finally, only one node remains on the stack: the root node of the pruning tree. This gives us the pruning tree we need.
**Algorithm 1** The process of pruning tree construction**Input:** postfix form of the logical expression *pfix*; empty stack *stack*; the value of the node *value*; child node *children*; the number of nodes in the subtree *num_children*; parent node *parent* **Output:** pruning tree
 1:**Class** ExpTree: //Constructing nodes in an expression tree 2:  3: **procedure** add_child(*children*): //Adding child nodes to a subtree 4:  **if** *children* is a list **then**: //The *children* is a list 5:   **for each** *ch* **in** *children* **do**: 6:    *ch.parent* ← **this** //Set the current node (**this**) to be the parent of the child *ch* 7:   **end for** 8:   append *children* to **this.children** 9:  **else**: //The *children* is just a node10:   *children.parent* ← **this** //Set the current node (**this**) to be the parent of *children*11:   append *children* to **this.children**12:**procedure** constructTree(*pfix*):13: **for** *i* **from** length of *pfix* −1 **to** 0 **step** −1 **do** //Iterate over *pfix* in reverse order14:  **if** isOperand(*pfix*[*i*]) **then** //*pfix*[*i*] is the operand15:   *node* ← **new** ExpTree(*pfix*[*i*]) //Use *pfix*[*i*] to create a new ExpTree node16:   push *node* to *stack*17:  **else:** //*pfix*[*i*] is the operator18:   *v1* ← pop from *stack* //*v1* is the current operand or subtree19:   *v2* ← pop from *stack* //*v2* is the subtree generated in the previous loop20:   *nn* ←**new** ExpTree(*pfix*[*i*]) //*nn* is the root node21:   **if** *v1.value* == *pfix*[*i*] **then** //*v1* is equal to the current operator *pfix*[*i*]22:    *nn.add_child*(*v1.children*) //Constructing the pruning tree23:   **else**:24:    *nn.add_child*(*v1*)25:   **if** *v2.value* == *pfix*[*i*] **then** //*pfix*[*i*] is equal to the operator of subtree *v2*26:    *nn.add_child*(*v2.children*) //Constructing the pruning tree27:   **else**:28:    *nn.add_child*(*v2*)29:   push *nn* to *stack*30: **end for**31:**return** *purning tree* ← pop from *stack*


### 3.3. Model Design

**Figure 4:**
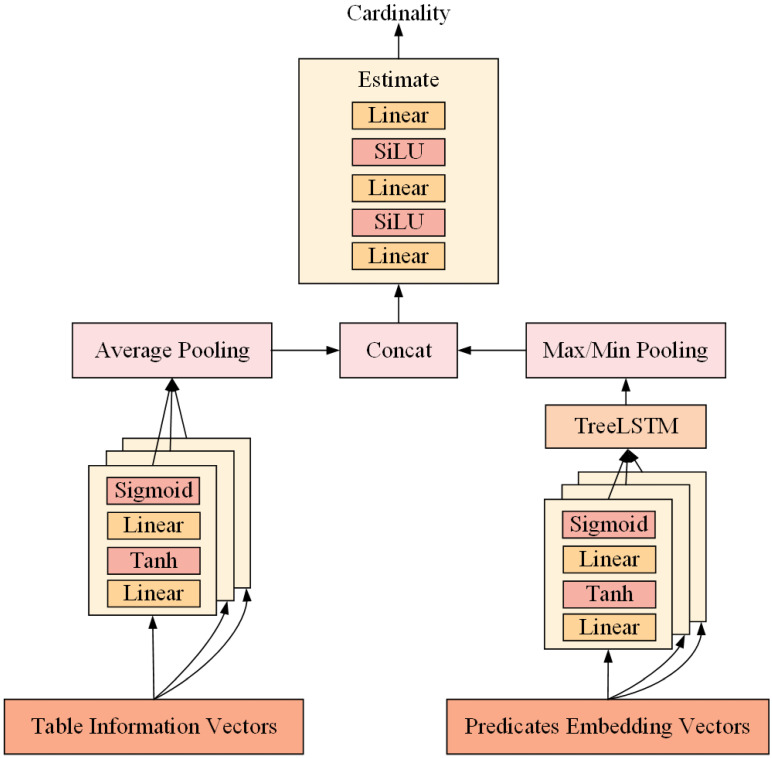
The overall framework of our cardinality estimator.

Standard neural network structures usually have difficulty efficiently handling vectors or matrices containing different information. To solve this problem, we divide a query statement into two parts in our study: the table name and the predicate. We use different neural networks to process these two parts to improve the expressiveness of the model. The overall framework of our model consists of three phases, as shown in Figure 4: the first stage is the MLP [37], which is used to process the table name information; the second stage involves passing the predicate information into the TreeLSTM neural network after it is processed by the MLP; and the third stage splices the vectors from the first two stages and transmits them to the MLP of the estimation layer to output the estimate of the cardinality.

All our MLPs are two-layer fully connected neural networks with tanh(*x*) = (exp(*x*) − exp(−*x*))/(exp(*x*) + exp(−*x*)) and sigmoid(*x*) = 1/(1 + exp(−*x*)) activation functions. Using tanh as the activation function for the first layer allows the network to map the input data nonlinearly while keeping the output in the range [−1, 1]. It helps the network better capture the input data’s nonlinear features and ensures a limited range of output values. Sigmoid is used as the second layer because our estimation results need to be scalars in the range [0, 1]. By combining tanh and sigmoid, the network can introduce certain nonlinear transformations in the first layer and then compress and probabilistically map the outputs in the second layer. This combination can help the network to achieve full expressive power without excessive complexity and is suitable for some moderate-complexity problems. The reason for not using the ReLU activation function is that ReLU outputs directly with positive input values and zero with negative input values. This results in some neurons that may never activate during training, known as the “neuron death” problem. The gradient in the positive region of ReLU is 1, which can lead to the gradient explosion problem, especially in deep networks with many layers. Gradient explosion may lead to unstable training and difficulty obtaining a converged model.

For the MLP in the estimation layer, we use a two-layer SiLU(*x*) = *x*/(1 + exp(−*x*)) activation function, where the derivatives of the SiLU function are continuous over the entire input range compared to ReLU. This smoothness can reduce the problem of vanishing gradients and make it easier to optimize the model during training. Moreover, the SiLU activation function introduces a nonlinear property that allows the MLP to learn more complex data patterns. By combining multiple layers of SiLU activation functions, the model can gradually learn higher-order feature representations, thus improving the expressive power of the model. These three stages are described in detail in the following section.

#### 3.3.1. Learning Table Information

In Section 3.2.1, we introduced the representation of table information. We concatenate the bitmap of the table name vector and the sampled tuples into a complete table information vector $V_{t}$ and then feed $V_{t}$ into an MLP for processing. To generalize the query features, we sum the outputs of all MLPs and average the results using averaging pooling. However, the dimensionality of the table information vector may not be consistent for each query. For example, query $q_{1}$ contains *n* tables, while query $q_{2}$ contains *n* + 1 tables. Therefore, the table information vector for query $q_{1}$ contains *n* row vectors, while the table information vector for query $q_{2}$ contains *n* + 1 row vectors. At this point, the dimensions of $q_{1}$ and $q_{2}$ do not match. To solve this problem, we use 0 to fill those row vectors with smaller dimensions so that all table information vectors have the same dimensionality. We use Equation (1) to represent the table name vector $w_{t}$ after MLP processing.

(1) $$w_{t}=\frac{\sum_{q\in Q}MLP\left(V_{t}\right)}{\left|T_{q}\right|}$$

#### 3.3.2. Learning Predicate Information

We were inspired by NLP [38], which mostly uses long short-term memory neural networks to process complex sentences. For the “WHERE” clause, we can consider it as a sentence. In order to better capture the semantic information implicit in complex clauses, we construct the “WHERE” clause as a tree structure and model the query using a tree-shaped long short-term memory neural network. The modeling of the tree structure allows us to deal with the semantic relations between Boolean connectives and predicates in a hierarchical way, and the TreeLSTM model can fully consider the information transfer between each predicate node and its children. This approach helps to improve the expressiveness of query statements and capture the intent and semantics of queries more accurately.

**Figure 5:**
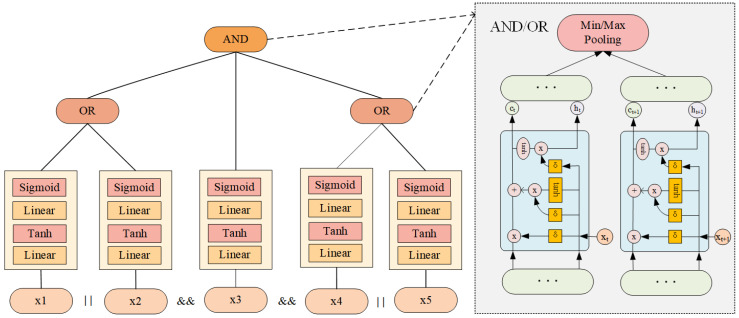
Working mechanism of our TreeLSTM model.

The traditional long short-term memory (LSTM) model is limited to only strictly sequential information propagation. In contrast, TreeLSTM is a neural network model for processing tree-structured data [38], an extended LSTM model approach for modeling and representing tree-structured data. Its basic idea is to extend LSTM units to a form applicable to tree-structured data. Traditional LSTM units are used for processing sequential data and can memorize the previous states and compute new states and outputs based on new inputs. However, each node may have multiple children for tree-structured data, so a more sophisticated mechanism is needed to handle this information. For this reason, TreeLSTM extends the LSTM unit by introducing two input gates and two output gates to accommodate the modeling of tree-structured data. In TreeLSTM, one set of input and output gates is used to process the information of the current node, while the other input and output gates are used to process the information for the child nodes. In this way, TreeLSTM can better capture the characteristics of the tree structure by considering the information for both the current node and its children. Using the TreeLSTM model, we can better capture the meaning and relationships of join operations in SQL queries, allowing us to better handle complex query statements and Boolean operators (conjunction or disjunction) between predicates. Specifically, we model the “WHERE” clause of the query statement as a tree structure based on the operators between the predicates, where the operators are the parent nodes and the predicates are the child nodes, as shown in Figure 3. Depending on the type of operator nodes, we select different TreeLSTM sub-networks and pass the child nodes (predicate nodes) into the TreeLSTM for processing, as shown in Figure 5.

Given a query statement, after constructing it as a tree structure, the transformation equations used in TreeLSTM are shown as Equations (2)–(8).

(2) $${\tilde{h}}_{j}=\sum_{k\in C(j)}h_{k}$$

(3) $$i_{j}=Sigmod\left(W^{\left(i\right)}x_{j}+U^{\left(i\right)}{\tilde{h}}_{j}+b^{\left(i\right)}\right)$$

(4) $$f_{jk}=Sigmod\left(W^{\left(f\right)}x_{j}+U^{\left(f\right)}h_{k}+b^{\left(f\right)}\right)$$

(5) $$o_{i}=Sigmod\left(W^{\left(o\right)}x_{j}+U^{\left(o\right)}{\tilde{h}}_{j}+b^{\left(o\right)}\right)$$

(6) $$u_{j}=Tanh\left(W^{\left(u\right)}x_{j}+U^{\left(u\right)}{\tilde{h}}_{j}+b^{\left(u\right)}\right)$$

(7) $$c_{j}=i_{j}⊙u_{j}+\sum f_{jk}⊙c_{k}$$

(8) $$h_{j}=o_{j}⊙Tanh\left(c_{j}\right)$$

where *C*(*j*) in Equation (2) denotes the set of children of node *j*, k∈C(j). $h_{k}$ is the hidden state of the kth child node, and ${\tilde{h}}_{j}$ is the summation of the information of all child nodes. $x_{j}$ in Equations (2)–(6) is the input, and $i_{j}$ and $o_{j}$ denote the input and output gates, respectively, computed using the aggregated information for the child nodes. The value of $i_{j}$ is close to 1 when the significant node is input and vice versa. In our study, an $i_{j}$ is a vector representation of an atomic predicate in a query statement and denotes element-by-element multiplication. In addition, one forgetting gate $f_{j_{k}}$ for each child node *k* is used in the TreeLSTM cell instead of a single forgetting gate, as in the standard LSTM.

#### 3.3.3. TreeLSTM Design

Figure 5 illustrates the working mechanism of our TreeLSTM model, which is divided into two stages from the bottom up.

In the first stage, the tree structure of the “WHERE” clause is input to the model through feature encoding. It is processed by a fully connected neural network and then output to the second stage.

In the second stage, the model selects the corresponding sub-network based on the operators (“AND” or “OR”) between atomic predicates during forward propagation. When dealing with compound predicates with multiple conditions, we need to learn the join relationships between the predicates and the distribution of the results after applying the predicates to the dataset. For compound predicates using the AND concatenation, we can estimate the number of outcomes satisfying the predicate from the minimum estimated number of outcomes satisfying the atomic predicate and, therefore, use min pooling to minimize the output of the upper layer. For compound predicates using the OR concatenation, we can estimate the number of results for the satisfying predicate from the maximum number of results that satisfy the atomic predicate and, therefore, use max pooling to obtain the maximum value for the output of the upper layer. The advantage of this treatment is that only leaf nodes need to be trained, thus enabling efficient batch training. Secondly, this model converges faster and performs better.

Overall, we use a two-layer MLP with tanh and sigmoid to process the predicate nodes. We use minimum pooling and maximum pooling instead of AND semantics and OR semantics for operator nodes, respectively. In the query shown in Figure 5, we can see the processing of each node and the final output. Equations (9)–(12) describe the forward propagation process of our model.

(9) $$\varphi \left(h_{k}\right)=\left\{\begin{array}{cc} \varphi_{\&\&}\left(h_{k}\right), & \mathrm{type}\left(j\right)=AND\\ \varphi_{\|}\left(h_{k}\right), & \mathrm{type}\left(j\right)=OR \end{array}\right.$$

(10) $${\tilde{h}}_{j}=\frac{1}{N(j)}\sum_{k\in C(j)}\varphi \left(h_{k}\right)$$

(11) $$w_{p}=Pooling\left({\tilde{h}}_{\mathrm{root}}\right)$$

(12) $$w_{out}=MLP\left(\left[w_{t},w_{p}\right]\right)$$

Equation (9) indicates that different types of sub-networks are selected according to the operator node. In Equation (10), *C*(*k*) represents the set of children of node *k*, and *N*(*k*) represents the number of children of node *k*. ${\tilde{h}}_{root}$ is a vector representation of the root node. $w_{t}$ is the table name vector mentioned in Section 3.3.1. MLP is the shallow neural network that undertakes the final cardinality estimation.

### 3.4. Loss Function

We construct a new loss function to overcome the problem of large and small cardinalities for the *Q-error*, as shown in (13). This loss consists of two parts: the *Q-error* and absolute value error. The *Q-error* is responsible for optimizing relative differences, and the absolute value error distinguishes between large and small errors. By combining the two types of loss, we can better adapt to various data distributions and improve the performance of the model. Equation (13) allows us to consider relative and absolute errors together to assess the accuracy of the cardinality estimation more fully.

(13) $$Loss=\alpha log\sum_{i=0}^{n}m\alpha x\left(\frac{{\hat{y}}_{i}}{y_{i}},\frac{y_{i}}{{\hat{y}}_{i}}\right)+\left(1-\alpha \right)\sum_{i=0}^{n}|{\hat{y}}_{i}-y_{i}|$$

**Figure 6:**
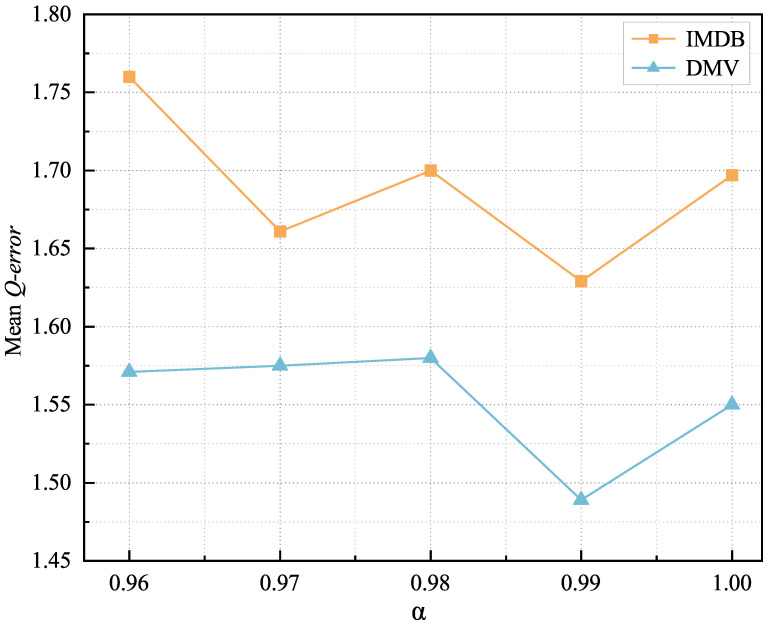
The impact of different α on the mean *Q-error* with different datasets.

Our final training goal is to minimize the loss function in Equation (13). To balance the effects of relative and absolute value errors, we introduce a balancing parameter α. We explored the effects of different values of α while ensuring that the relative error remains dominant in the total loss function. We conducted a series of comparative experiments, as described in Section 4.4, and the experimental results are shown in Figure 6. In Figure 6, we can see that the best performance with our model was obtained when α = 0.99. α = 0.99 indicates that the relative error term is 99% of the overall loss function, while the absolute value error term is 1%. The aim is to ensure that the relative error term dominates the total loss function in order to fit the data distribution better and add a penalty score in cases of significant errors. Such a weighting configuration helps us pay more attention to the optimization of the relative error during training and provides finer control over the performance of the model.

## 4. Evaluation

### 4.1. Datasets

This study used two datasets with different characteristics for multi-table and single-table experiments.

1. The IMDB dataset [3]. The IMDB dataset is a dataset from the Internet Movie Database (IMDB), one of the largest movie and television databases. The dataset contains 21 tables, and after joining all the tables in a certain order, it has a data volume of 2.8 × $10^{14}$. Therefore, many researchers use this dataset or several tables in this dataset to study multi-table related content, and we chose six tables for our experiments.
2. The DMV dataset [12]. The DMV dataset is a large, single-table dataset for storing car, sled, and boat registration information. The dataset includes the personal information of car and boat owners, basic information about vehicles and boats, and registration times. The dataset has a data volume of 1.25 × $10^{7}$, and some columns were selected for use in our experiments.

### 4.2. Evaluation Metrics

A commonly used evaluation metric in cardinality estimation is the *Q-error* defined in Equation (14) [39], which is used to measure the difference coefficient between the true and the estimated cardinality. However, the *Q-error* cannot distinguish between large and small cardinalities. This problem suggests that the *Q-error* only considers the relative error between the true and estimated cardinalities and does not consider the effect of the absolute error. Therefore, our study also uses the evaluation metrics commonly used in regression problems, which consider the absolute error: the mean absolute error (*MAE*), shown in Equation (15), and the symmetric absolute percentage error (*SMAPE*), shown in Equation (16). Here, ${\hat{y}}_{i}$ denotes the estimated value, $y_{i}$ denotes the true value, and *n* is the sample size.

(14) $$Q-error=max\left(\frac{y_{i}}{{\hat{y}}_{i}},\frac{{\hat{y}}_{i}}{y_{i}}\right)$$

(15) $$MAE=\frac{1}{n}\sum_{i=1}^{n}\left|{\hat{y}}_{i}-y_{i}\right|$$

(16) $$SMAPE=\frac{100\%}{n}\sum_{i=1}^{n}\frac{\left|y_{i}-{\hat{y}}_{i}\right|}{\left(\left|y_{i}\right|+\left|{\hat{y}}_{i}\right|\right)/2}$$

### 4.3. Comparison Methods and Experimental Environment

We chose to use two traditional databases in our comparison (i.e., PostgreSQL 9.15.19 and MySQL 5.7.26) that use histograms to estimate cardinality on a sampled basis. However, histogram methods usually make a simplifying assumption of independence between columns. This assumption of independence helps to decompose the joint distribution. However, the actual situation often violates this assumption, especially in the case of complex query statements, and the estimation results may be poor.

In addition, we compared three learning-based approaches. The first one was the MSCN [7], which is a multiset convolutional neural network showing that deep learning models can learn complex interactions between predicates and even capture the cross-correlation of columns of connections. However, the limitation of this approach is that deep neural networks with average pools have difficulty representing complex structures and do not have semantic extraction capabilities. This approach may lead to large errors for complex or tree-structured query plans. Therefore, the MSCN is difficult to generalize to support complex SQL queries and cannot handle string-type data. In order to make it able to handle string data types, we modified it so that it could train the query statements we generated. Another approach is the VSCNN [40], a vertical scanning convolutional neural network for processing query statements and extracting their semantic information. However, this model uses the same encoding method for Boolean operators and predicates when processing query statements, ignoring the unique role of Boolean operators. This approach greatly impacts the estimation results when dealing with complex query conditions. Finally, TreeRNN [35], a neural network based on a recursive tree structure, can handle query statements containing Boolean operators but only single-table queries. In addition, this method uses one-hot encoding for string data. When there are many different values for the operands, one-hot encoding causes data sparsity and dimensional disaster problems, leading to a sharp increase in feature dimensionality and the consumption of a large amount of storage space and computational resources, as well as easily leading to overfitting problems.

We trained our model by using the PyTorch framework and CUDA. All our experiments were conducted using a computer with an Intel(R) Core(TM) i7-10750H CPU, 16 GB of memory, 512 G SSD, and a Windows 10 operating system. Our equipment was manufactured by Hewlett-Packard, located in Chongqing, China. It is worth noting that, during the experimental process, all our experiments involved training, validation, and testing with two datasets, the IMDB and DMV datasets, respectively. During training, validation, and testing, we kept all conditions consistent, such as the experimental environment, evaluation metrics, and so on.

### 4.4. Hyperparameter Tuning

To optimize the performance of our model, we need to adjust the number of epochs, the batch size, the number of hidden units, the learning rate, and the parameter α in the loss function. Both the learning rate and the batch size affect the convergence behavior during model training. More hidden units mean a larger model size and increased training and prediction costs, with the benefit of allowing the model to capture more data.

In order to obtain the appropriate hyperparameters, we made the following attempts, as shown in Table 2. After continuous training and testing, our model achieved the best performance when we used a learning rate of 0.001, α = 0.99, 100 epochs, a batch size of 32, and 128 hidden units. Also, we used the early stopping method to avoid the overfitting problem. Moreover, we compared several gradient descent algorithms and found that Adam [41] performed the best, so the Adam gradient descent algorithm was used in our study.

Figure 6 shows the variation in the mean *Q-error* for different values of the balance parameter α in the loss function. It can be seen that the best performance for our model was obtained when α = 0.99.

### 4.5. Model Complexity

For model complexity, we studied in detail the memory occupied by the model, the average execution time per query, and the number of references using the IMDB dataset. Table 3 shows that our model used about 1.6 MB of memory, less than the MSCN and VSCNN methods. In addition, the average execution time for each query was slightly longer than with the MSCN and VSCNN. This shows that our model maintained high performance while being lightweight.

### 4.6. Estimation Quality

**Figure 7:**
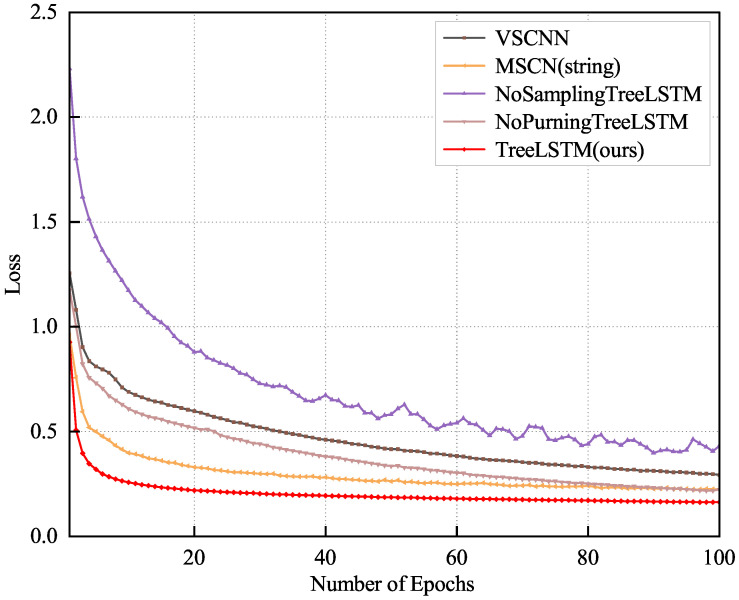
Convergence of the loss function for the IMDB dataset with the number of epochs.

**Figure 8:**
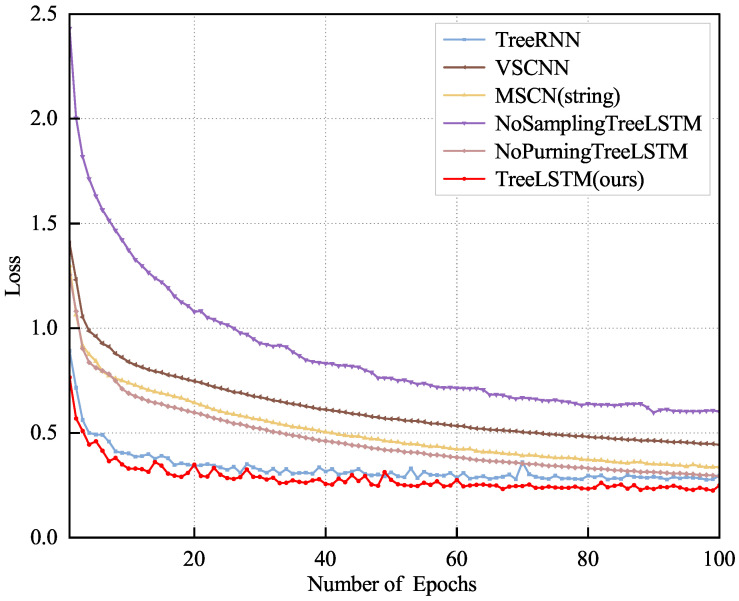
Convergence of the loss function for the DMV dataset with the number of epochs.

In our experiments, we used 80,000 queries as the training set, 10,000 as the validation set, and the remaining 10,000 queries as the test set. Figure 7 and Figure 8 show the results for our model for both the IMDB and DMV datasets compared with other methods with the validation set, illustrating the better results and convergence performance of our proposed TreeLSTM model.

Figure 7 shows that the VSCNN converged the slowest and TreeLSTM converged the fastest and performed the best with the multi-table dataset. Moreover, after adding the sampling method, the effect and convergence speed of TreeLSTM were better than the NoSamplingTreeLSTM, which indicates that the effect and convergence speed of the model could be improved after the sampling method was applied because introducing the sampling method allowed our model to learn the data distribution better. In addition, TreeLSTM is more effective than the NoPurningTreeLSTM because the pruning simplifies the structure of the SQL tree, which allows our model to learn the hidden semantic information in the query statements faster and accelerate the convergence speed. Figure 8 shows that our model still worked best with the single-table dataset. This is because our model is based on the idea of NLP, so it can better handle the semantics implied in query statements with both multi-table and single-table datasets. Since TreeRNN can only handle single-table queries, we only compared it using the DMV dataset.

Table 4 shows the average *Q-error*, *MAE*, and *SMAPE* results for our model for different cardinality estimators using the test set of the IMDB dataset, with the best results highlighted in bold. We can see that the *Q-error*, *MAE*, and *SMAPE* for TreeLSTM were better than with the other methods. The average *Q-error* for our model was much smaller than the average *Q-error* for the traditional database because traditional cardinality estimation methods perform cardinality estimation based on the assumption of column independence, which can easily overestimate or underestimate the cardinality value, leading to slow or unpredictable query performance. The problem becomes more complex as the number of predicate columns increases, resulting in an optimal execution that cannot be obtained. Compared to other learning-based methods for cardinality estimation, our method exhibited significantly better performance, with average *Q-error*, *MAE*, and *SMAPE* values that were 1.2, 2.0, and 1.69 times smaller than those of the MSCN and 1.9, 2.1, and 3.1 times smaller than those of the VSCNN, respectively. This significant improvement was due to the strong semantic recognition capability of our model. Moreover, the average *Q-error*, *MAE*, and *SMAPE* of our method were 6.4, 4.9, and 4.7 times smaller than those of the NoSamplingTreeLSTM, respectively, because the inclusion of the sampling method allowed our model to use the information from the samples in the base table to learn the distribution of the data. Furthermore, the average *Q-error*, *MAE*, and *SMAPE* of our method were 1.5, 1.8, and 1.7 times smaller than those of NoPurningTreeLSTM, respectively, because, by adding the pruning algorithm, we could simplify the SQL tree we constructed, making it easier for the model to learn semantics in the query statements and improving the accuracy of the estimation.

Table 5 shows a comparison of the average *Q-error*, *MAE*, and *SMAPE* for different cardinality estimators with the test set of our model for the DMV dataset. Most of the values of the metrics in Table 4 are smaller than those in Table 5 because the DMV dataset is a single-table database that does not involve SQL connections. In contrast, the IMDB dataset is a multi-table database containing more SQL connections. The average *Q-error* of our model was still much smaller than the mean *Q-error* of the cardinality estimation methods in traditional databases. Compared with several learning-based methods, the average *Q-error*, *MAE*, and *SMAPE* of our method were 2.0, 2.6, and 1.2 times smaller than those of the MSCN, respectively; 2.0, 2.8, and 1.5 times smaller than those, respectively; and 1.1, 1.7, and 1.1 times smaller than those of TreeRNN, respectively. Additionally, the average *Q-error*, *MAE*, and *SMAPE* of our method were 6.0 times, 4.7 times, and 2.4 times smaller than those of NoSamplingTreeLSTM and 1.6 times and 2.3 times smaller than those of NoPurningTreeLSTM, respectively.

The experimental results show that our proposed model can handle single-table and multi-table databases well. It can identify the relationships between different join operations well because it constructs the query statements as a tree structure and encodes the predicates using word embedding. By using sampling methods, our model can make full use of the sample information in the base table to learn the distribution of the data, thus improving the performance of the model.

Overall, our TreeLSTM model performed well with both datasets, which indicates that our model has good generalization abilities and can estimate new query cardinalities. As shown by the experimental results, traditional database systems tended to overestimate the cardinalities with a large *Q-error*. Compared with traditional estimators, the learning-based cardinality estimator can greatly reduce the estimation error because the learning-based model can learn a large number of SQL features from the training data.

### 4.7. Prediction Time Comparison

**Figure 9:**
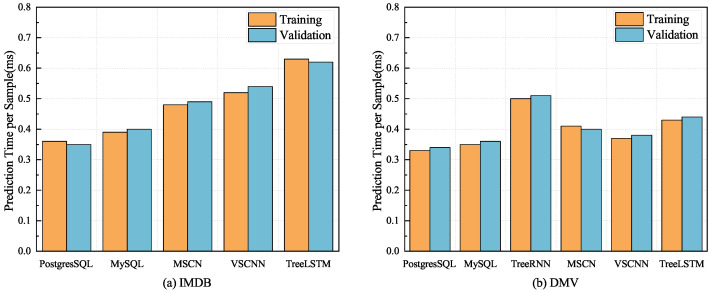
Prediction time comparison for different methods with the IMDB and DMV datasets.

Figure 9 compares the prediction times between different methods with different datasets. In Figure 9a, we can see that the prediction times of the TreeLSTM with the multi-table IMDB dataset were around 0.63 ms and 0.62 ms per sample for the training and validation sets. Traditional cardinality estimation methods have shorter prediction times because they use sampling-based methods, which estimate the cardinality of the entire dataset by randomly sampling a small portion of the data to predict the query execution time. The prediction times of the MSCN and VSCNN were slightly lower than that of TreeLSTM because our model requires query statements to be constructed with a tree structure, resulting in a longer prediction time. However, these prediction times are acceptable relative to the performance improvement of our models. Figure 9b shows the prediction times of our model with the single-table dataset. The prediction times of all methods with the DMV dataset were shorter than those with the IMDB dataset. As the single-table dataset does not involve SQL joins, and the model does not have to learn table-to-table relationships, the prediction times of all methods were reduced with the DMV dataset. TreeRNN’s prediction time was longer than all other methods because TreeRNN uses one-hot encoding to encode the predicates, leading to a longer prediction time because of the larger dimensionality of the one-hot vector.

## 5. Conclusions

In this study, we solve the cardinality estimation problem by introducing a tree long short-term memory neural network model that constructs SQL queries into a tree structure and converts them into tree vectors. Then, TreeLSTM is used to capture the relationships between tables and between predicates. It performs better than other traditional and learning-based methods in handling single-table and multi-table queries and can handle string-type predicates. In addition, we introduce the sampling method, which helps the model to learn more information from the tables and improves the accuracy of the cardinality estimation. Moreover, we construct a new loss function to overcome the drawback that the *Q-error* cannot distinguish between large and small cardinalities. Finally, we conducted extensive experiments with the real IMDB and DMV datasets, and the experimental results showed that the proposed TreeLSTM model significantly improves the accuracy of cardinality estimation compared with both traditional and learning-based cardinality estimation methods.

We will continue to improve our model in order to apply it to more queries. Currently, our model cannot adapt to updated environments. When a large amount of data are inserted or deleted, it is difficult for our model to keep track of these changes, so it needs to be retrained, which is time-consuming. We will design a better approach to solve these two problems in the future.

## Figures and Tables

**Table 1:** Summary of work related to cardinality estimations.

Category	Method	Advantage	Disadvantage
Traditional cardinality estimation methods	Summary-based	Simple to implement and widely used	Based on independence assumptions with large errors
	Sampling-based	More accurate estimation, connection-oriented estimation for join queries	Requires extra space to store sampled data; has 0-tuple problem
Learning-based cardinality estimation methods	Query-driven	The most accurate and fastest cardinality estimation method, excellent performance with multiple tables	Requires extensive training; insufficient generalization ability
	Data-driven	Accurate and relatively stable estimates with a single table	Requires extensive training; insufficient generalization ability
	Hybrid	Performs well with a single table and estimates accurately	It cannot be trained directly on the data and would increase the cost of the model

**Table 2:** Hyperparameter settings for experiments.

Epochs	Batch Size	Hidden Units	Learning Rate	α
100	32	32	0.0001	0.96
200	64	64	/	0.97
/	128	128	/	0.98
/	256	256	/	0.99
/	/	/	/	1.00

**Table 3:** Complexity study for the proposed model.

Method	Memory	Time	Parameters
MSCN	2.6 MB	0.48 ms	683,521
VSCNN	5.2 MB	0.52 ms	1,379,841
TreeLSTM (ours)	1.6 MB	0.63 ms	409,106

**Table 4:** *Q-error*, *MAE*, and *SMAPE* of different cardinality estimators with the IMDB dataset.

Method	*Q-Error*	*MAE*	*SMAPE*
PostgreSQL	2 × $10^{5}$	/	/
MySQL	2 × $10^{5}$	/	/
VSCNN	3.12	2291	0.59
MSCN (string)	1.94	2176	0.31
NoSamplingTreeLSTM	10.37	5387	0.89
NoSamplingTreeLSTM	2.38	1959	0.33
TreeLSTM (ours)	1.63	1109	0.19

**Table 5:** *Q-error*, *MAE*, and *SMAPE* of different cardinality estimators with the DMV dataset.

Method	*Q-Error*	*MAE*	*SMAPE*
PostgreSQL	1 × $10^{5}$	/	/
MySQL	1 × $10^{5}$	/	/
TreeRNN	1.71	1526	0.40
VSCNN	3.01	2593	0.55
MSCN (string)	2.96	2418	0.43
NoSamplingTreeLSTM	8.90	4374	0.86
NoSamplingTreeLSTM	2.36	2106	0.33
TreeLSTM (ours)	1.49	923	0.36

## Data Availability

The datasets used in this study are all public datasets.
